# MGPT: A Multi‐task Graph Prompt Learning Framework for Drug Discovery

**DOI:** 10.1002/advs.202506444

**Published:** 2025-06-25

**Authors:** Yang Li, Youhan Sun, Xinyu Qin, Xin Gao, Guohua Wang

**Affiliations:** ^1^ College of Computer and Control Engineering Northeast Forestry University Hexing Road Harbin Heilongjiang 150040 China; ^2^ Computer Science Program, Computer, Electrical and Mathematical Sciences and Engineering Division King Abdullah University of Science and Technology (KAUST) Thuwal 23955‐6900 Kingdom of Saudi Arabia; ^3^ Center of Excellence for Smart Health (KCSH) King Abdullah University of Science and Technology (KAUST) Thuwal 23955‐6900 Kingdom of Saudi Arabia; ^4^ Center of Excellence on Generative AI King Abdullah University of Science and Technology (KAUST) Thuwal 23955‐6900 Kingdom of Saudi Arabia; ^5^ School of Computer Science and Technology Harbin Institute of Technology Harbin Heilongjiang 150001 China

**Keywords:** drug associations, heterogeneous graph network, multi‐task prompt tuning, self‐supervised contrastive learning

## Abstract

Predicting accurate drug associations, including drug‐target interactions, drug side effects, and drug‐disease relationships, is crucial in biomedical research and precision medicine. Recently, the research community is increasingly adopted graph representation learning methods to investigate drug associations. However, translating advancements in graph pre‐training to the domain of drug development faces significant challenges, particularly in multi‐task learning and few‐shot scenarios. A unified Multi‐task Graph PrompT (MGPT) learning model is proposed providing generalizable and robust graph representations for few‐shot drug association prediction. MGPT constructs a heterogeneous graph network using different entity pairs as nodes and utilizes self‐supervised contrastive learning of sub‐graphs in pre‐training. For downstream tasks, MGPT employs learnable functional prompts, embedded with task‐specific knowledge, to enable robust performance across a range of tasks. MGPT demonstrates the ability of seamless task switching and outperforms competitive approaches in few‐shot scenarios. MGPT emerges as a robust solution to the complexities of multi‐task learning and the challenges associated with limited data in drug development.

## Graphical abstract

MGPT is a unified multi‐task graph prompt learning model providing generalizable and robust graph representations for few‐shot drug association prediction. MGPT demonstrates the ability of seamless task switching and outperforms competitive approaches in few‐shot scenarios. MGPT emerges as a robust solution to the complexities of multi‐task learning and the challenges associated with limited data in drug development.

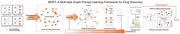

## 1 Introduction

The diverse associations in pharmacology, such as drug‐target interactions (DTI), drug‐disease associations, drug side effects, and drug chemical properties, are essential for advancing drug development,^[^ ^1^ ^]^ personalizing medicine, and understanding treatment efficacy. Identifying drug‐target interactions, for instance, aids in understanding drug mechanisms, streamlining target identification, and expediting drug design. Predicting potential side effects is crucial for drug safety, enabling early detection of adverse reactions and modification of drug candidates. Accurately predicting these drug associations can improve drug discovery efficiency, reduce the costs of failed trials, and advance personalized medical care by tailoring treatments to individual patient characteristics, thereby revolutionizing the pharmaceutical industry. The integration of bioinformatics, pharmacology, and computational modeling has become increasingly vital in the era of big data and artificial intelligence for precision medicine and new drug discovery.^[^ ^2^ ^]^

The research landscape in drug association predictions has experienced a paradigm shift, with various methods being used to uncover complex relationships in the pharmaceutical field. Key methodological trends include machine learning and graph‐based approaches, such as support vector machines (SVM), random forests, convolutional neural networks (CNN), have been widely applied. These techniques leverage diverse datasets encompassing molecular structures, biological pathways, and clinical information to discern complex patterns and predict drug‐target interactions,^[^ ^3^, ^4^, ^5^ ^]^ drug side effects,^[^ ^6^, ^7^ ^]^ and drug disease associations. F. Napolitano, et al.^[^ ^8^ ^]^ explored drug repositioning using a machine‐learning approach, incorporating chemical structure similarity, target similarity, and gene expression similarity into a support vector machine (SVM). J. Peng, et al.^[^ ^9^ ^]^ proposed DTI‐CNN, employing Jaccard similarity coefficients and a restart random walk model with a convolutional neural network for DTI prediction. S. Dey, et al.^[^ ^10^ ^]^ employed a chemical fingerprint algorithm to transform drugs into graphical structures, compressed through convolution, and used a fully connected neural network to predict drug‐side effect associations. H. Luo, et, al.^[^ ^11^ ^]^ proposed a computational method based on the assumption that similar drugs are often associated with similar diseases and vice versa. This approach utilized integrated similarity metrics and the BiRW (Biased Random Walk) algorithm to identify potential new indications for a given drug.

In recent years, graph neural network methods have seen extensive application in bioinformatics due to their ability to learn rich topological information and biological data.^[^ ^12^, ^13^ ^]^ Graph convolutional neural networks (GCN) and graph attention networks (GAT) have been utilized to model relationships between drugs, targets, and diseases. Y. Luo, et al.^[^ ^14^ ^]^ introduced the “guilt‐by‐association” concept, proposing the DTINet model that captures complex relationships in drug discovery through a heterogeneous network and network representation algorithms. Zhao et al.^[^ ^15^ ^]^ established the GCN‐DTI model, utilizing a graph convolutional neural network for predictive learning. Unlike most existing methods, GCN‐DTI doesn't separately construct drug and target networks; instead, it considers the interactions between drugs and protein pairs (DPP). Another model, proposed by J. Peng, et al.,^[^ ^16^ ^]^ is the EEG‐DTI model, which takes a heterogeneous network input containing various biological entities like drugs, proteins, diseases, and side effects. It employs a graph convolutional neural network to achieve end‐to‐end information prediction. B. Hu, et al.^[^ ^17^ ^]^ introduced a method for predicting drug‐related side effects using a heterogeneous network that integrates various interaction data. They represented the correlations between drugs and side effects as a network graph, with each node's representation synthesized from its adjacent nodes. P. Xuan, et al.^[^ ^18^ ^]^ developed heterogeneous graphs combining drug‐disease associations with medicinal chemical substructures. It integrated both specific and common topologies along with pairwise attributes of drugs and side effects. Z. Gao, et al.^[^ ^19^ ^]^ utilized three views to construct a similarity network between drugs and diseases. Employing two graph encoders, both local and global topological structures are concurrently modeled. Subsequently, a graph contrastive learning approach is applied to collaboratively train node representations, thereby enhancing the quality of predictions.

Despite the significant contributions that the above‐mentioned methods have made to drug association research, they still have some shortcomings. First, all these methods require a significant amount of training data. However, in the field of drug development, obtaining large‐scale annotated data is both expensive and time‐consuming.^[^ ^20^ ^]^ Second, how do we ensure the accuracy of downstream tasks with few‐shot learning? Machine learning often thrives on large‐scale data, and its performance can suffer significantly when confronted with limited samples.^[^ ^21^ ^]^ Third, most existing research on predicting drug association is focused on individual tasks, lacking a unified framework to integrate multiple tasks.^[^ ^22^, ^23^, ^24^ ^]^ An effective approach to these challenges is the “pre‐training and prompt‐tuning” schema, where a model is pre‐trained on related tasks with abundant data and then prompt‐tuned on a downstream task of interest. While pre‐training models have been effective in language and vision domains, as well as in graphs,^[^ ^25^, ^26^, ^27^, ^28^ ^]^ it remains an open question how to effectively use pre‐training in drug association research.

To address the aforementioned challenges, we present MGPT (Multi‐task Graph PrompT), a unified learning framework for few‐shot drug association prediction that integrates graph‐based representation learning with prompt‐based task adaptation. MGPT constructs a heterogeneous graph in which each node denotes a concatenated entity pair (e.g., drug–protein, drug–disease), encompassing diverse biomedical entities such as drugs, proteins, and diseases. This graph is pre‐trained via a self‐supervised contrastive learning strategy to encode both structural and semantic similarities across entity pairs. In the subsequent prompt‐tuning phase, a learnable task‐specific prompt vector is introduced to incorporate prior knowledge captured during pre‐training. Serving as a semantic anchor, the prompt enables effective knowledge transfer and rapid adaptation to downstream tasks under limited supervision. By leveraging the learned representations of entity pairs, MGPT facilitates few‐shot learning across multiple drug‐related tasks, including drug‐target interaction prediction, drug‐side effect association, and drug‐disease relationship inference. We evaluate MGPT on two benchmark drug association datasets and compare its performance against a range of competitive baseline models. Results show that MGPT consistently outperforms existing approaches, achieving state‐of‐the‐art performance in few‐shot settings. In particular, MGPT surpasses the strongest baseline, GraphControl, by over 8% in average accuracy, demonstrating its superior generalization and robustness in low‐resource scenarios. To further elucidate the model's cross‐task transferability, we analyze the similarity of learned prompt vectors using cosine similarity. We observe notably high similarity scores among pharmacologically related tasks, especially between drug‐side effect interaction and drug substitution, suggesting that MGPT captures shared semantic structures critical for effective multi‐task learning. Collectively, these findings highlight MGPT as a powerful and generalizable framework for few‐shot learning across diverse drug association tasks. Given the promising results of MGPT in few‐shot scenarios, we believe it would be worthwhile to explore its application in real‐world settings where data is often limited. This could include domains such as drug discovery, where obtaining large labeled datasets is often challenging due to the cost and time required for experimental validation. MGPT's ability to effectively learn from limited data could potentially speed up the discovery process by enabling more efficient use of resources and reduced dependence on large datasets.

## 2 Results

### 2.1 Overview of MGPT Model

The Multi‐task Graph Prompt (MGPT) Learning model is a cutting‐edge framework developed for few‐shot drug association prediction. This process begins with the construction of a heterogeneous graph network, wherein nodes are entity pairs created by combining different node types such as proteins, drugs, and diseases. The model then undergoes self‐supervised contrastive learning to pre‐train these graph nodes based on their similarities. In specific downstream tasks, MGPT utilizes a learnable prompt vector. This vector incorporates pre‐trained knowledge to semantically represent the tasks, aiding in few‐shot learning across various tasks like predicting drug‐target interactions, drug side effects, and drug‐disease relationships. The MGPT Framework stands out as a sophisticated and specialized approach for consolidating information across diverse tasks in bioinformatics.

### 2.2 MGPT Outperforms the Baseline Methods Under the Few‐Shot Learning Condition in all the Downstream Tasks

To evaluate the performance of MGPT in six downstream tasks, including predicting the interactions of drug‐chemical structure, drug‐drug side effect, drug‐drug substitute, drug‐target protein, target protein‐gene ontology, we initially conducted a comparison with seven state‐of‐the‐art methods in graph representation learning (detailed in Supporting Information I) on two datasets, detailed experimental settings are available in the “Methods” section. These methods spanned supervised learning, unsupervised learning, and a combination of pre‐training, prompting, and fine‐tuning. Here's an overview of each approach: Supervised learning based methods trained a Graph Neural Network (GNN) using nodes and edges in a graph to learn node representation vectors, which were then directly applied for model inference. We selected well‐established supervised learning baselines, including GCN, GAT, and GraphSAGE. Unsupervised learning based methods didn't rely on labeled node data or predefined outcomes. It enabled the model to autonomously uncover hidden structures or patterns in the data, thus acquiring node information for use in downstream tasks. We included DGI, an approach to contrastive learning that maximized the mutual information between local and global representations of nodes in the graph. For specific downstream tasks, the model used the node information to make predictions, training a Multi‐Layer Perceptron (MLP) for this purpose. Pre‐training, prompting, and fine‐tuning methods typically involved training a Graph Neural Network model with a self‐supervised task. Prompts were used to bridge the gap between pre‐training and downstream tasks, followed by fine‐tuning the pre‐trained model on downstream tasks. We considered the GPPT model, which pre‐trained the Graph Neural Network using a masked edge prediction task to learn graph node representations. Special tokens were designed to prompt the model, which was then fine‐tuned for downstream tasks. In addition, we included GCC, a self‐supervised graph pre‐training framework that generalizes across diverse graph structures by discriminating subgraph instances within and across datasets, enabling the learning of transferable structural representations. We also evaluated GraphControl, a ControlNet‐inspired deployment module that conditionally integrates task‐specific information during fine‐tuning or prompt tuning by aligning input spaces and incorporating target‐specific attributes, thereby enhancing model adaptability and accelerating convergence on attributed graphs. Both methods share conceptual alignment with our framework in their use of pre‐training for transferable graph representation learning and task‐specific adaptation.

MGPT outperformed all the baselines across all tasks in the few‐shot setting (refer to **Table** **1** and **Table** **2**). Specifically, MGPT showed significant improvements over the baselines, achieving over a 8% increase in average performance on two datasets compared to the most competitive baseline method, GraphControl. The results indicated that MGPT could effectively capture interactions between drugs and other entities, which is crucial in drug design and modification. Furthermore, it also proved the effectiveness of MGPT in pre‐training and prompting, highlighting its ability to learn structural information and content within graphs, and transfer the knowledge to downstream tasks through prompting.

**Table 1:** The comparison results of MGPT and the baselines in Luo's dataset under the few‐shot (1% samples) learning scenario (meaning that there were 1% training samples during the prompt‐tuning).

Method	Luo's data				
	All	Drug–protein	Drug–disease	Drug‐side effect	Protein‐disease
GCN	50.83	52.78	50.23	52.00	50.04
GAT	49.97	49.65	50.33	49.96	50.46
GraphSAGE	50.21	49.91	50.18	52.83	49.94
GPPT	49.95	50.02	50.07	50.27	49.97
DGI	51.09	51.61	52.50	53.57	53.75
GCC	54.19	49.84	56.39	58.08	62.83
GraphControl	55.80	53.15	61.54	56.63	61.08
MGPT	**65.17**	**54.95**	**74.20**	**72.98**	**77.12**

**Table 2:** The comparison results of MGPT and the baselines in Zheng's dataset under the few‐shot (1% samples) learning scenario.

Method	Zheng's data					
	All	Drug‐chemical	Drug‐side effect	Drug‐substituent	drug‐target	Target‐go
GCN	50.13	52.83	51.81	52.30	51.53	50.16
GAT	50.19	50.03	49.62	50.09	49.88	50.96
GraphSAGE	50.35	50.02	49.96	49.93	50.05	49.92
GPPT	50.26	53.05	53.06	56.61	51.29	50.16
DGI	51.06	52.87	55.30	52.00	53.66	51.41
GCC	54.58	50.33	51.13	50.49	50.93	54.36
GraphControl	60.52	65.33	65.93	69.30	69.90	59.79
MGPT	**70.10**	**70.06**	**70.07**	**77.78**	**75.44**	**65.46**

Interestingly, we observed that two graph pre‐training methods, GPPT and DGI, performed well in certain tasks. Apart from MGPT, GPPT achieved state‐of‐the‐art performance on Zheng's dataset, while DGI excelled on Luo's dataset. This suggests that graph pre‐training methods can effectively capture relationships between entities and enhance performance in downstream tasks. However, MGPT still maintained a lead in various downstream tasks compared to these methods. For GPPT, its more complex prompting strategy might not have achieved the expected results in a few‐shot setting, and its fine‐tuning strategy had limited modifications to the model itself. As for DGI, the lack of a connection between self‐supervised models and downstream tasks meant that merely training a new MLP for predictions did not allow the model to effectively gain prior knowledge in few‐shot scenarios. It is worth noting that, aside from MGPT, GraphControl achieved the strongest overall performance by dynamically injecting task‐specific cues during the fine‐tuning process, thereby effectively alleviating the transferability‐specificity dilemma. Across both benchmark datasets, it almost outperformed GPPT and DGI across all evaluated tasks. However, some supervised models did not perform well in few‐shot scenarios. The complexity of the model, combined with the few‐shot mode, might not facilitate effective learning, particularly without appropriate prompting to transfer the acquired knowledge to downstream tasks.

### 2.3 Performance of MGPT in Different Settings of Few Shot Learning

In order to explore the performance of MGPT in different downstream tasks including predicting the interactions of drug‐chemical structure, drug‐drug side effect, drug‐drug substitute, drug‐target protein, target protein‐gene ontology, with a small amount of training data, we conducted an evaluation of the performance of the MGPT under various few‐shot learning configurations on two established benchmark datasets (**Figure** **2**a and **Figure** **3**a). We set *k*% values to 1%, 3%, 5%, 7%, and 10%, meaning that there were *k*% training samples during the prompt‐tuning. We observed that the model demonstrated strong performance in predicting drug substituents and drug target, even with minimal values of *k*%, achieving a quasi‐elimination rate exceeding 75%. It suggested that these two tasks were relatively simple, and pre‐training can capture sufficient information without extensive prompt‐tuning for downstream tasks. This observation provided valuable insights for the effective application of drug pre‐training models in downstream tasks. The task that was most sensitive to the number of prompt‐tuning samples was drug‐chemical structure prediction. This sensitivity indicated that the drug chemical structure task demanded a high level of accuracy and precision. A larger number of samples could provide more information, thereby enhancing the model's ability to capture patterns and nuances in this specific task.

**Figure 1:**
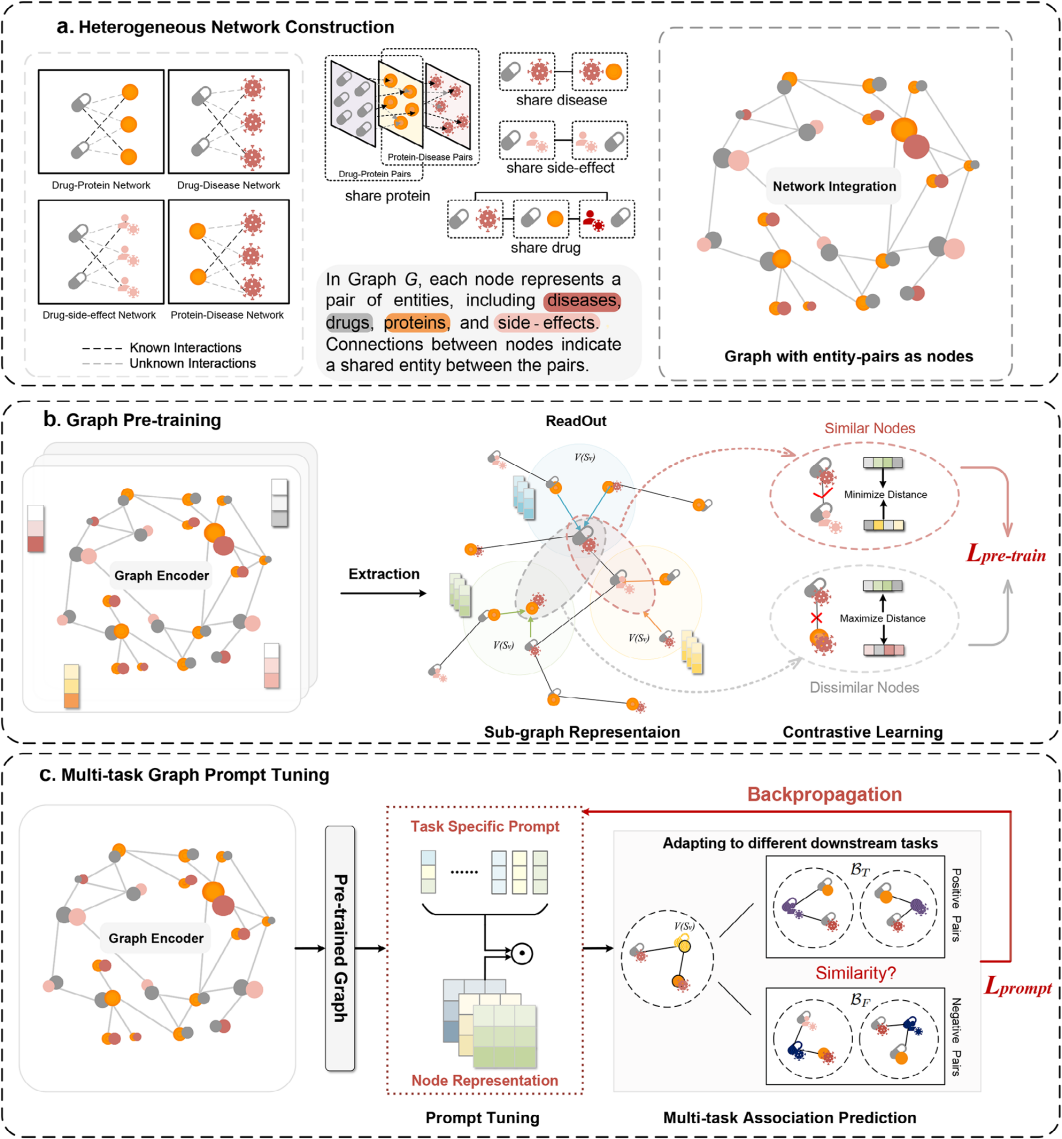
An illustrative diagram of MGPT. a) Heterogeneous Network Construction: Entities such as drugs, proteins, diseases, and side effects are concatenated into node pairs, laying the groundwork for a heterogeneous network. b) Graph pre‐training utilizes sub‐graph similarity contrastive learning, where nodes sharing entities exhibit a higher scientific basis for similarity. c) Multi‐task Prompt Learning. During this stage, learnable prompt vectors are introduced for downstream tasks, serving as parameters for the readout operation, facilitating the use of diverse aggregation functions on the sub‐graphs specific to each task.

**Figure 2:**
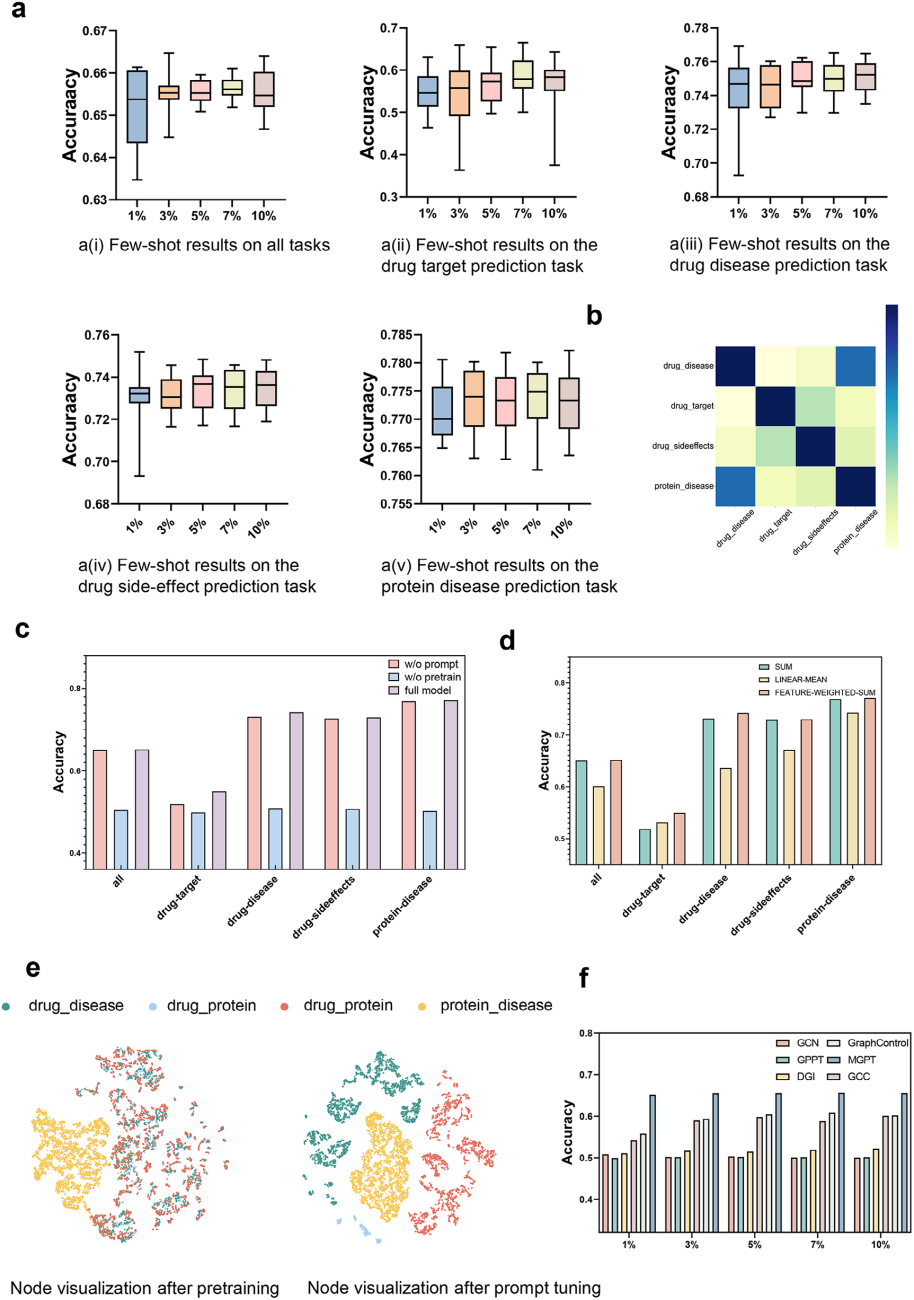
Overall performance of MGPT in Luo's dataset. a) Few‐shot (*k*% samples) learning results of MGPT on downstream tasks using Luo's dataset. b) Heatmap illustrating the similarity of prompt vectors across variousdownstream tasks in Luo's dataset. c) Ablation study results for MGPT using Luo's dataset. d) Analysis of various readout strategies in MGPT using Luo's dataset. e) Visualization of node representations corresponding to different task types, conducted during pretraining without the use of prompts using Luo's dataset and pretraining with the inclusion of prompts using Luo's dataset. f) Comparison of MGPT with state‐of‐the‐art (SOTA) methods in the few‐shot (*k*% samples) learning scenario using Luo's dataset.

**Figure 3:**
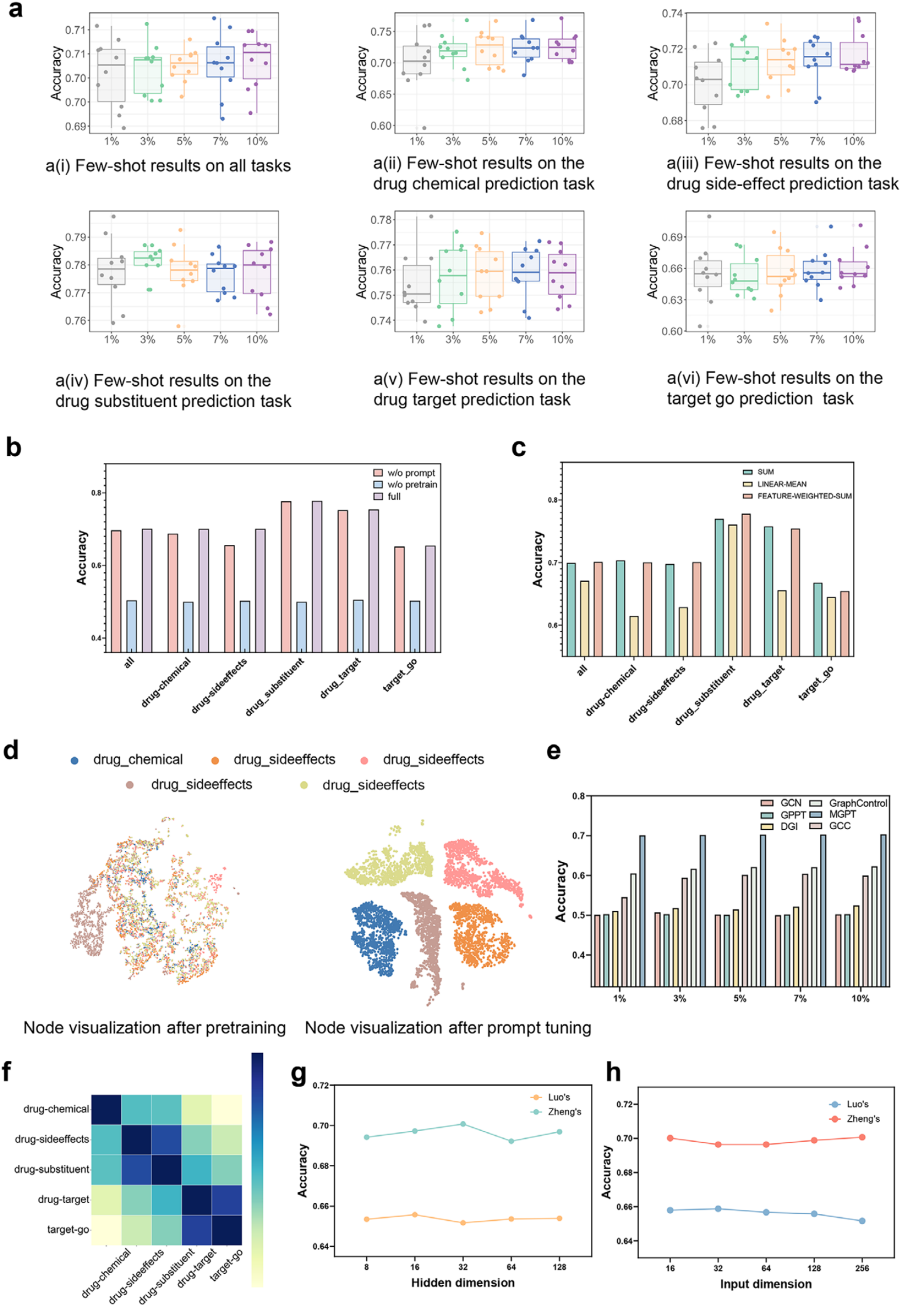
Overall performance of MGPT in Zheng's dataset. a) Few‐shot (*k*% samples) learning results of MGPT on downstream tasks using Zheng's dataset. b) Ablation study results for MGPT using Zheng's dataset. c) Analysis of various readout strategies in MGPT using Zheng's dataset. d) Visualization of node representations corresponding to different task types, conducted during pretraining without the use of prompts using Zheng's dataset and pretraining with the inclusion of prompts using Zheng's dataset. e) Comparison of MGPT with state‐of‐the‐art (SOTA) methods in the few‐shot (*k*% samples) learning scenario using Zheng's dataset. f) Heatmap illustrating the similarity of prompt vectors across various downstream tasks in Zheng's dataset. g) Parameter sensitivity analysis for different hidden dimensions: 8, 16, 32, 64, and 128. h) Parameter sensitivity analysis for different input dimensions: 16, 32, 64, 128 and 256.

Simultaneously, we conducted evaluations of MGPT and other prominent methods using two datasets in the different settings of few‐shot learning scenario, presenting the average prediction results for all tasks (Figure 2f and Figure 3e). It can be observed that MGPT significantly outperformed the other methods comprehensively. With the increase of the *k*% value, the performance of most methods improved. However, MGPT maintained its advantages throughout the range of *k*% values. The experimental results also demonstrated that, in general, MGPT performed better when there were more samples available for training. However, even with a limited number of samples, MGPT could still achieve good results.

### 2.4 MGPT Discovers New Drug Targets

MGPT has demonstrated notable potential in identifying new drug targets. For example, it predicted an interaction between nortriptyline (NT) and the multidrug transporter P‐glycoprotein (P‐gp), despite contradicting the negative ground‐truth label. Remarkably, this prediction aligns with findings from recent experimental research,^[^ ^29^ ^]^ which investigated how NT interacts with P‐gp to influence brain concentrations of psychotropic drugs.

To further support the model's prediction, we conducted molecular docking using AutoDock Vina,^[^ ^30^, ^31^ ^]^ which yielded a binding energy of –7.2 kcal/mol for the NT‐P‐gp complex, indicating a favorable interaction. Structural analysis via the PLIP^[^ ^32^ ^]^ revealed that NT forms a stable complex with P‐gp, involving seven hydrophobic interactions and four hydrogen bonds (**Figure** **6**). These interactions suggest a strong binding affinity between the two molecules.

Moreover, in vivo experiments reported in Ref. [29] demonstrated that pre‐administration of Cyclosporine A, a known P‐gp inhibitor, significantly increased the brain/blood ratio of NT in rats. This implies that P‐gp plays a critical role in regulating NT concentration in the brain, thereby supporting the biological relevance of the interaction predicted by MGPT.

### 2.5 Ablation Studies on MGPT

To comprehensively analyze the effectiveness of each component of MGPT, we conducted two ablation experiments: *MGPT without prompt* to evaluate the impact of our prompt strategy, and *MGPT without pretrain* to evaluate whether the pre‐training stage effectively acquired prior knowledge (Figure 2c, Figure 3b). The following observations were made: (*i*) The *full model* MGPT consistently achieved the highest performance, showcasing the necessity of the pre‐training and prompt strategy, regardless of the absence of pre‐training or prompt modules. (*ii*) Without pre‐training, the model's performance was substantially compromised, highlighting the critical nature of unsupervised data pre‐training and validating the potential of leveraging graph pre‐training frameworks for drug association predictions.

To better leverage prompts for guiding downstream tasks, we explored the effects of different readout strategies in MGPT, including SUM, LINEAR‐MEAN, and FEATURE‐WEIGHTED‐SUM. These strategies showed varying impacts across tasks. It became evident that FEATURE‐WEIGHTED‐SUM was effective for all tasks, while the SUM strategy was more suitable for target‐go prediction. In contrast, the performance of the LINEAR‐MEAN strategy is relatively weaker compared to the other two strategies. It suggested the possibility of customizing specific guidance strategies for downstream tasks or adopting more cost‐effective general strategies (Figure 2d and Figure 3c).

We next evaluated the parameter sensitivity in MGPT. The hidden dimensions were adjusted to 8, 16, 32, 64, and 128 layers (Figure 3g). For Zheng's dataset, encompassing numerous downstream tasks, optimal performance was achieved with 32 dimensions. In contrast, Luo's dataset, with fewer downstream tasks, showed better results with 16 dimensions.

We also conducted a sensitivity analysis of the node input dimensions (Figure 3h). The node dimension set to 32 resulted in the highest performance for Luo's dataset, while a dimension of 256 was most effective for Zheng's dataset. Importantly, changes in node dimension had only a slight effect on accuracy, highlighting the robustness of MGPT.

### 2.6 Investigating the Prompt Learned by MGPT

In this section, our objective is to delve into the learning mechanisms of MGPT, particularly focusing on the role of prompts in both pre‐training and prompt‐tuning phases for various downstream tasks. Our approach includes a detailed analysis of how different task types are represented within the network's node structure, and how this representation is influenced by the use of prompts.

Our investigation began with the visualization of node representations corresponding to different task types within the network, conducted in two phases: pretraining without prompts and pretraining with prompts (Figure 2e for Luo's dataset and Figure 3d for Zheng's dataset). Initially, we visualized node representations learned without prompts, establishing a baseline. This visualization revealed the natural segregation or grouping of tasks within the network's architecture (see Figure 2e). The subsequent phase, illustrated in Figure 2e, entailed visualizing nodes after prompt‐based pre‐training. This step enabled us to assess the impact of prompts on node representation. Comparison analysis indicated that the nodes visualized in Figure 2e, influenced by prompts, showed a clearer differentiation between task types. This distinct separation is significant, suggesting that prompts substantially enhance MGPT's ability to differentiate various task types. Similar conclusions were also observed in Zheng's dataset (Figure 3d). The improved distinction in task types facilitated by prompts has significant implications for MGPT's functionality. It implies that prompts act as a guiding tool, enabling MGPT to transition more effectively between downstream tasks. This flexibility is essential for MGPT's application in diverse real‐world scenarios, where rapid and accurate task switching is essential. Overall, these results highlight the pivotal role of prompts in enhancing MGPT's task‐specific learning, solidifying its utility in a range of downstream tasks. The visual evidence from our node analysis further emphasizes the importance of prompts in refining and directing MGPT's learning trajectory.

**Figure 4:**
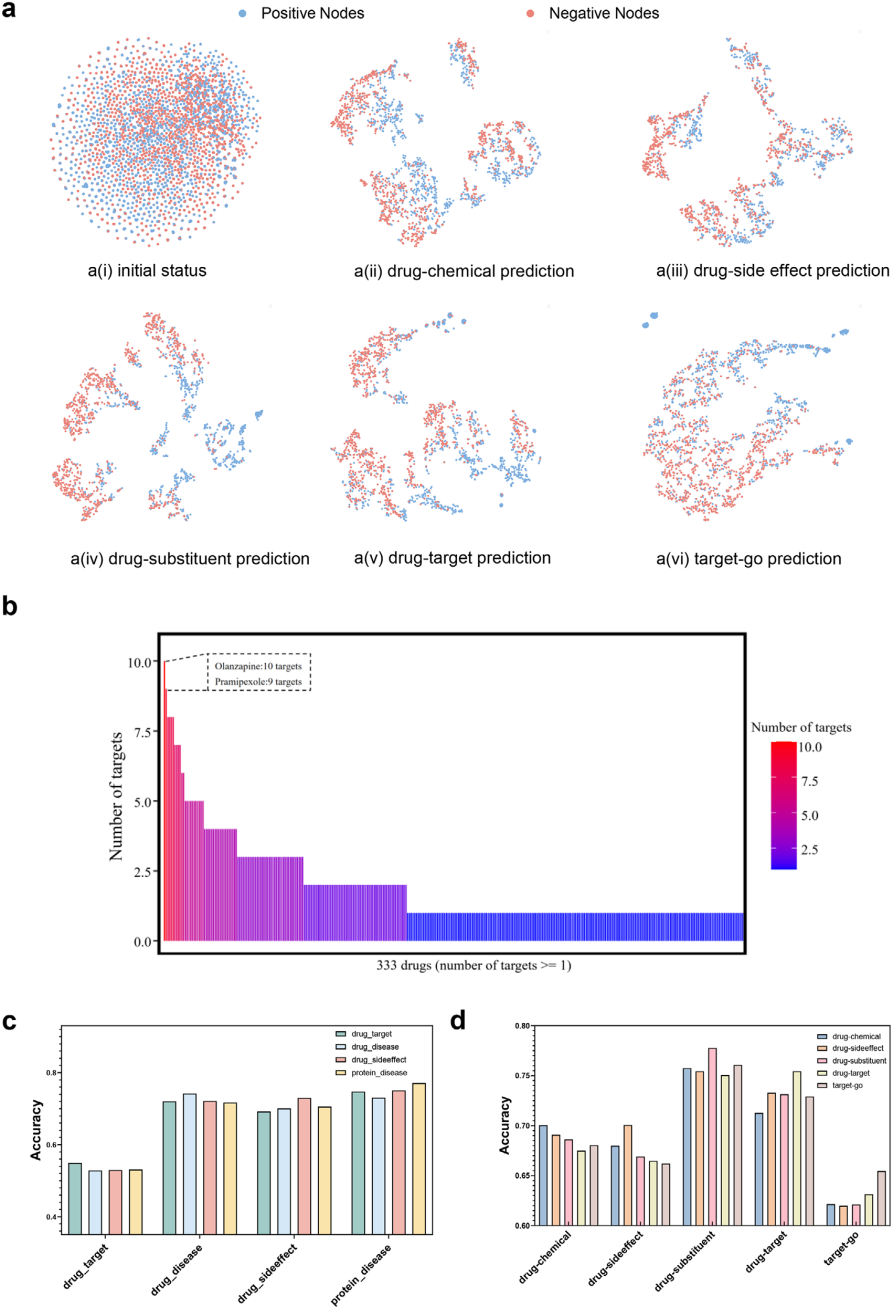
Visualization of the role played by prompt in decision boundaries and domain adaptation. a) Visualization of the decision boundaries of the downstream tasks. b) Distribution of the number of drug targets. The top three drugs with the highest number of targets are Quetiapine, and Pramipexole. c) Performance of prompt domain adaptation in Luo's dataset. d) Performance of prompt domain adaptation in Zheng's dataset.

To better understand the effectiveness of prompt‐tuning in downstream tasks with pre‐training hints, we utilized t‐SNE visualization to scrutinize the distribution of sample vectors across various downstream tasks within two datasets, specifically under the condition of a few‐shot (1% samples) learning scenario (**Figure** **4**a). We represented negative nodes with the number 0 and the color red, while positive nodes were denoted as 1 and colored blue. In Zheng's dataset, our model effectively identified the decision boundaries in all five downstream tasks, compared to the initial vector distribution. Notably, in the drug‐substituent task diagram, the decision boundary was more apparent than in other tasks, and this clarity corresponded to the highest predictive accuracy among the downstream tasks. This observation indicated that using t‐SNE to visualize the representation of node pairs accurately reflected the performance of the model.

We further evaluated the applicability of MGPT's prompt vectors in downstream tasks by performing a cosine similarity analysis (Figure 2b and Figure 3f). The results revealed some notable patterns in the similarity of task specific prompt vectors across different tasks. The results indicate that prompt vectors of the same type generally exhibit higher similarity. For example, in the Luo's dataset, the protein‐disease and drug‐disease vectors, as well as the target‐go and drug‐target vectors in the Zheng's dataset, show this pattern. The prominence of these similarities was an important insight, as it reflected the inherent relatedness in the nature of these tasks. It suggested that these particular task areas shared common features, which the MGPT model was able to effectively identify and capture. On the other hand, the prompt vectors for other downstream tasks exhibited significantly lower levels of similarity. The variation was particularly significant as it showcased the prompt vectors' capacity to effectively distinguish between various tasks. This ability to differentiate is essential when utilizing pre‐trained models, allowing for the customized application of pre‐trained knowledge. Specific adaptation to the unique features of each task ensures that the model can be prompt‐tuned effectively to address the distinct requirements and characteristics of different tasks.

### 2.7 Negative Sampling Strategies in Pretraining

During pretraining, we adopt a contrastive learning framework, wherein the selection of positive and negative samples plays a critical role in determining the quality of the learned representations. This section evaluates three commonly used strategies for negative sample selection in graph‐based contrastive learning:

Strategy 1: Community‐Aware Negative Sampling This approach applies a community detection algorithm to partition nodes into distinct communities. Negative samples are selected from nodes outside the target node's community, thereby reducing the likelihood of selecting false negatives‐nodes that are semantically similar yet incorrectly treated as dissimilar.

Strategy 2: Degree‐Based Stratified Negative Sampling Nodes are grouped into degree‐based strata. Negative samples are drawn either from different degree tiers or from distant nodes within the same tier. To further mitigate false negatives, nodes with lower degrees are sampled with higher probability, as they are less likely to be functionally similar to the target node.

Strategy 3: Random Negative Sampling This baseline method selects negative samples randomly from nodes not directly connected to the target node. While simple and computationally efficient, it lacks semantic awareness.

**Figure 5:**
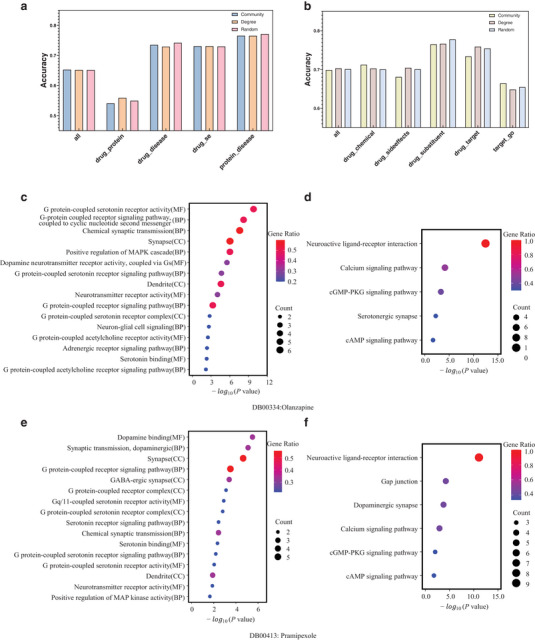
Comparative analysis of negative sampling strategies in contrastive learning and enrichment analysis of three typical drug targets. a) Performance comparison of different negative sampling strategies in contrastive learning on luo's dataset. b) Performance comparison of different negative sampling strategies in contrastive learning on Zheng's dataset. c) GO enrichment analysis of genes linked to drug Quetiapine. d) KEGG enrichment analysis of genes linked to drug Quetiapine. e) GO enrichment analysis of genes linked to drug Pramipexole. f) KEGG enrichment analysis of genes linked to drug Pramipexole.

As shown in **Figure** **5**a,b, the three strategies exhibit slight variations in downstream task performance. However, the overall differences are marginal, likely due to the scale of the graph enabling the model to learn robust representations regardless of the sampling strategy. These results suggest that the model exhibits a degree of robustness to negative sampling design choices.

**Figure 6:**
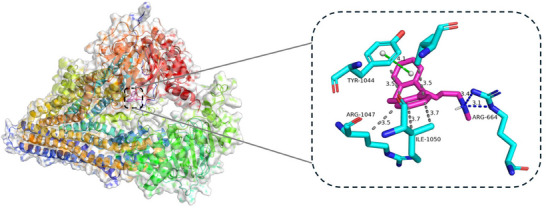
The interactions between nortriptyline and P‐glycoprotein profiled by PLIP.^[^ ^32^ ^]^

### 2.8 Exploring Domain Adaptation Using Prompts in MGPT

To evaluate the domain adaptation capability of prompts in MGPT, we conducted experiments where a prompt from one task was used to guide predictions in different tasks (Figure 4c,d). The findings indicated that, in Luo's dataset, prompts learned from protein‐disease and drug‐disease interactions exhibited enhanced domain transfer guidance for various tasks. In Zheng's dataset, the prompt learned from drug‐substituent interactions demonstrated a more pronounced domain adaptation ability. Overall, these results confirm that prompts learned by MGPT can be effectively used for domain adaptation between downstream tasks, offering valuable insights for domain transfer learning in drug association analysis.

### 2.9 Results of Enrichment Analysis of Target Genes

We analyzed the drugs with the top three highest numbers of targets as predicted by MGPT, focusing on Quetiapine, and Pramipexole of which are psychotropic drugs (Figure 4b). Quetiapine is utilized for managing bipolar disorder, schizophrenia, and major depressive disorder. Pramipexole is utilized for managing Parkinson's disease and restless legs syndrome. It is a dopamine agonist that helps alleviate symptoms such as stiffness, tremors, muscle spasms, and poor muscle control associated with Parkinson's disease. The enrichment analysis of GO and KEGG after integration of the genes linked to the predicted targets were shown in (Figure 5) and Supporting Information Table S4. The GO enrichment analysis demonstrated that the targets are significantly related to Neuron‐glial cell signaling, postsynaptic membranes, and neuronal cell bodies ‐ all crucial to emotional processing (Figure 5c,e). The KEGG pathway enrichment analysis showed that these targets were notably enriched in pathways including neuroactive ligand‐receptor interaction, calcium signaling pathway, and the serotonergic synapse pathway, as detailed in (Figure 5d,f).

## 3 Discussion

This article presents a unified Multi‐task Graph PrompT (MGPT) learning framework tailored for various few‐shot drug association prediction tasks. By leveraging a heterogeneous graph network and employing self‐supervised contrastive learning during pre‐training, MGPT effectively addresses the challenges of multi‐task learning in the context of drug development. The integration of a learnable prompt vector in the prompt‐tuning stage enhances semantic task representation, enabling efficient few‐shot learning across diverse tasks such as drug‐target interactions, drug‐side effects, and drug‐disease relationships. The evaluation against various benchmarks on two datasets demonstrates the robustness and effectiveness of the MGPT framework. It exhibits exceptional task‐switching capabilities and consistently outperforms competitive approaches, achieving an average improvement of over 8%. Case Study demonstrated that MGPT can identify previously unexplored associations, such as novel drug targets.

We conducted a comprehensive analysis of the MGPT prompt vector. By visualizing the node representations for different task types, with and without the task prompt vector, we discovered that MGPT, through its pre‐training and prompt‐tuning mechanism, can effectively differentiate downstream task types, thus promoting the rapid switching among these tasks. The cosine similarity analysis of prompt vectors further emphasized the model's proficiency in identifying similarities between related tasks, as well as its capability to discern differences among various types of tasks. This property is very important for the effective application of the pre‐training model to a wide range of downstream tasks, which can ensure that each task can benefit from the most relevant and specially customized pre‐training knowledge. Additionally, the domain adaptation experiment demonstrated that the prompts learned from MGPT can be effectively utilized for domain adaptation between downstream tasks, offering valuable insights for transfer learning in drug association analysis. Finally, by visualizing the classification boundaries of each downstream task before and after pre‐training, we discovered that MGPT's pre‐training effectively learns the inherent semantic information of nodes and uncovers the implicit relationships within the network structure. It leads to notable improvements in the few‐shot classification of downstream tasks.

Despite its strong empirical performance, the MGPT framework has several limitations that warrant further exploration. A primary concern is its lack of interpretability. Although the integration of prompt vectors improves task discrimination and downstream performance, the semantic meaning of these vectors remains opaque, limiting our ability to understand the model's decision‐making process. To address this, future work could incorporate explainable AI techniques, such as attention mechanisms or gradient‐based attribution methods, to shed light on how prompts influence model behavior. Additionally, MGPT currently represents pairwise entity relationships as individual nodes within the graph. While this design simplifies modeling, it may fail to capture the complexity of higher‐order or multi‐relational biological interactions, potentially constraining the framework's expressiveness in more intricate biomedical settings. Overall, while the MGPT framework shows strong potential for few‐shot drug association prediction, further refinement and expansion will be essential to fully realize its applicability to real‐world biomedical challenges, particularly within the dynamic and data‐scarce landscape of modern drug discovery.

## 4 Conclusion

MGPT represents a significant advancement in addressing the critical challenge of limited data and multi‐task integration in drug association prediction. By leveraging a unified multi‐task graph learning framework, MGPT constructs a heterogeneous graph network using entity pairs and employs self‐supervised contrastive learning to pre‐train robust graph representations. This novel pre‐training strategy, combined with learnable functional prompts that incorporate task‐specific knowledge, enables seamless task switching and achieves state‐of‐the‐art performance in few‐shot learning scenarios. Comprehensive experiments across multiple downstream tasks, including drug‐target interactions, drug‐side effects, and drug‐disease relationships, demonstrate MGPT's superior ability to generalize and adapt to diverse tasks with minimal annotated data. Moreover, its ability to effectively utilize limited samples makes it particularly valuable in real‐world applications where data scarcity is a common challenge, such as drug discovery and personalized medicine. By reducing reliance on large‐scale annotated datasets, MGPT accelerates the drug development process, enhances prediction accuracy, and provides actionable insights for precision medicine. This framework not only advances computational methods in pharmacology but also paves the way for more efficient and data‐efficient approaches in biomedical research.

Beyond the current scope, the proposed framework holds strong potential for extension to other critical areas of drug discovery, such as drug property classification and toxicity prediction, where labeled data is typically scarce. Future work may focus on adapting MGPT to incorporate a broader range of biomedical entities and relational types, or on integrating domain‐specific knowledge sources to further enhance predictive performance. Pursuing these directions could significantly expand the applicability of MGPT and contribute to the development of more robust and comprehensive computational tools for pharmacological research.

## 5 Experimental Section

### MGPT Architecture

MGPT consists of three primary components: Heterogeneous Network Construction, Graph Pre‐training, and Multi‐task Prompt Learning (**Figure** **1**). In the first stage, we build a heterogeneous network by aggregating data from four distinct tasks into node pairs. These nodes are linked based on a specific rule: nodes sharing the same biological substance are connected, creating a network interlinking drugs and their related entities. The next phase is graph pre‐training, where we use contrastive learning to analyze node similarities. Following this, we developed a trainable prompt vector, and with a limited dataset, directed the pre‐trained model toward specific downstream tasks. In the final step, we execute drug association prediction tasks using the learned node representations and the prompt.

### MGPT Architecture‐Heterogeneous Network Construction

We formally define a heterogeneous graph as G = (V,E,A,R), characterized by a node type mapping function ϕ:V→A, and an edge type mapping function ψ:E→R. Here, V represents node set, E denotes edge set, A represents node types, and R represents edge types. It's essential to highlight that each node v∈V and each edge e∈E are specifically associated with specific types in A and R, respectively. This signifies that ϕ(v)∈A and ψ(e)∈R. Additionally, heterogeneous graphs consist of various node and edge types, with the requirement |A|+|R|>2 for their definition.

We first concatenate drugs with other entities such as targets, side effects, and diseases to form entity pairs, which are treated as nodes in the heterogeneous graph G, such as *v* ^(*drug*, *target*)^. These entity pair nodes are undirected and unweighted, and they are sampled randomly from all possible associations present in the dataset. A subset of these entity pairs is selected based on task relevance and data balance considerations, as detailed in the Experimental Setup section. This construction allows us to reformulate the downstream association prediction tasks as classification problems over entity pairs.^[^ ^3^, ^15^ ^]^ Specifically, our heterogeneous network includes four types of entity pair nodes, including drug‐protein, drug‐disease, drug‐side effect, and protein‐disease pairs. When constructing the edges in the heterogeneous network, given the hypothesis that if two entity pair nodes *v* ^(*a*, *b*)^ and *v* ^(*a*, *c*)^ share any common component entity *a*, then these two entity pair nodes are more likely to share similar features, and we connect them with an edge. Note that, in the process of establishing connections in the heterogeneous graph, we did not use any annotated data.

### MGPT Architecture‐Graph Pre‐Training

During the pre‐training phase, we design a low‐cost task that doesn't require biological experiments or annotated data based on the presence of connections between two nodes. Generally, connected nodes exhibit higher similarity compared to unconnected nodes, as shown in Figure 1. To transfer the knowledge learned in the pre‐training stage to downstream node classification, we adopt the graph pre‐training method based on sub‐graph similarity contrastive learning.

Given the heterogeneous graph G=(V,E,A,R), our pre‐training objective is to train a function: $f:V\to R^{d}$ that maps each node v∈V to a *d*‐dimensional vector representation, where *d* ≪ |*V*|. These learned vectors should encapsulate both node features and structural information, facilitating downstream tasks, particularly the node classification problem. A natural idea is to use Graph Neural Networks (GNN) to learn the function.

Node Representation: Following a strategy that adheres to GNN spatial message passing, we learn node representations through recursive aggregation. For instance, at the *k*‐th layer, the representation of node *v* is defined as follows:

(1) $$h_{v}^{k}=\mathrm{AGGREGATE}\left(h_{v}^{k-1},\left\{h_{u}^{k-1}:l\in N_{v}\right\};\theta^{k}\right)$$

where $h_{v}^{k}\in R^{d}$ represents the representation vector of node *v* in the *k*‐th layer. Initially, $h_{v}^{0}$ contains the features from the original input. Θ, serving as the set of learnable parameters in GNN, can be expressed as Θ = {θ^1^, θ^2^, …}. Exactly, the AGGREGATE function is crucial in incorporating information from neighboring nodes $h_{u}^{k-1}$, where $u\in N_{v}$, into the representation of the central node *v* during the aggregation process. The specific form of this function determines how information is combined and updated across the node's neighborhoods. Specifically, we use the AGGREGATE function proposed by GIN:^[^ ^33^ ^]^

(2) $$h_{v}^{k}={\mathrm{MLP}}^{k}\left(\left(1+\varepsilon^{k}\right)\cdot h_{v}^{k-1}+\sum_{u\in N_{v}}h_{u}^{k-1}\right)$$

where ε is a learnable parameter or a fixed scalar.

Sub‐graph Representation: In a heterogeneous graph G=(V,E,A,R), we generally define the sub‐graph for node *v* as follows $S_{v}=\left(V\left(S_{v}\right),E\left(S_{v}\right)\right)$, where V and E represent sets of nodes and edges, as given by the following formula:

(3) $$\begin{array}{ccc} V\left(S_{v}\right) & = & \{d(u,v)\leq \delta |u\in V\},\\ E\left(S_{v}\right) & = & \left\{\left(u,u^{\prime }\right)\in E|u\in V\left(S_{v}\right),u^{\prime }\in V\left(S_{v}\right)\right\} \end{array}$$

where δ represents the scope of the subgraph *S*_*v*_ as defined, and *d*(*u*, *v*) denotes the distance between nodes *u* and *v* within the graph. The subgraph *S*_*v*_ aggregates information from node *v* and its neighbors within the δ range, facilitating a smoother transition of pre‐training tasks to downstream tasks.

To compute subgraphs, a readout operation is employed to aggregate the representations of node *v* and its neighbors within the subgraph. The READOUT function is defined as:

(4) $$s_{v}=\text{READOUT}\left(\left\{h_{u}:u\in V\left(S_{v}\right)\right\}\right)$$

In this specific case, the READOUT function SUM is defined as:

(5) $$s_{v}=\left(h_{v}+\sum_{u\in V(S_{v})}h_{u}\right)$$

Self‐supervised Contrastive Learning: For an entity pair node *v* in our graph G, we choose two nodes, *m* and *n*. While node *m* is directly connected to node *v*, there is no direct connection between node *n* and node *v*. Our objective is to reduce the association between the subgraph of node *v* and the subgraph of node *n* while enhancing the association between the subgraph of node *v* and the subgraph of node *m*. Note that when constructing the heterogeneous graph, edges are only established between nodes if they share a common entity. Therefore, nodes that share entities have a higher scientific basis for similarity. To achieve this, we define our contrastive loss function in the pre‐training stage as follows:

(6) $$L_{\text{pre-train}}\left(\Theta \right)=-\sum_{\left(v,m,n\right)\in V_{pre}}\ln\frac{\exp(sim\left(s_{v},s_{m}\right)/\tau )}{\sum_{c\in \{m,n\}}\exp\left(sim\left(s_{v},s_{c}\right)/\tau \right)},$$

where $V_{pre}$ represents the randomly selected training set, *sim* is the similarity between the two sub‐graphs, Θ denotes the GNN parameters, and τ is the temperature parameter. The optimal $\Theta =\arg\min_{\Theta }L_{\text{pre-train}}\left(\Theta \right)$ obtained at this stage will serve as the weight for the downstream task model, with the aim of transferring the learned graph knowledge to downstream tasks.

### MGPT Architecture‐Multi‐Task Prompt Tuning

In the new drug development stage, the available samples are typically limited. Hence, the capability of few‐shot learning is crucial for drug research and development. Additionally, drawing inspiration from NLP prompts, we've crafted a highly informative and trainable prompt for multi‐task drug‐related associations. The objective is to guide the pre‐trained model toward our downstream tasks, enhancing its ability to leverage prior knowledge.

Prompt Design: Graph prompts differ significantly from language prompts due to the following reasons. First, the format of prompts varies: in natural language processing, prompts take the form of textual instructions for downstream tasks, while in our tasks, they are represented graphically. This makes designing graph prompts more challenging than language prompts as we must consider not only the content but also the structural information of the graph. Second, while prompts in natural language processing are often manually crafted, manual prompt creation in graph processing is impractical.

Current research on graph prompts is limited, with few studies applying graph prompts to drug association prediction. Furthermore, most existing graphs are homogeneous, and the design of prompts for heterogeneous graphs has not been explored. Additionally, there is a scarcity of research on multi‐task learning. To address these issues, we propose learnable prompts specifically designed for drug association prediction at the graph level. These prompts integrate both content and structural information, employing diverse readout strategies for different tasks.

To enable flexible adaptation across multiple downstream tasks, we introduce a learnable prompt vector **p** that acts as a task‐specific control signal during the readout phase. In contrast to conventional feature embeddings, this vector serves as a lightweight, differentiable instruction that guides the aggregation of subgraph representations in a task‐aware manner. Conceptually, the prompt vector conditions the model to attend to task‐relevant features during representation extraction, thereby facilitating precise and efficient multi‐task generalization. For instance, the prompt readout operation for a specific task, such as drug‐target interaction, is as follows:

(7) $$s_{v}^{\left(d-p\right)}=\text{READOUT}\left(\left\{p^{\left(d-p\right)}⊙h_{u}:u\in V\left(S_{v}\right)\right\}\right),$$

Here, $s_{v}^{\left(d-p\right)}$ signifies the subgraph representation of node *v* in a particular task, drug‐protein interaction (*d* − *p*). The symbol ⊙ represents element‐wise multiplication. There is a diverse range of readout options available, and specific READOUT schemes can be applied based on different node categories to enhance the model's performance. Specifically, we design different READOUT strategies, including SUMMATION, AVERAGING, and FEATURE‐WEIGHTED‐SUM.^[^ ^34^ ^]^ In this specific case, the READOUT function FEATURE‐WEIGHTED‐SUM is defined as:^[^ ^34^ ^]^

(8) $$s_{v}=\left(h_{v}+\sum_{u\in V(S_{v})}h_{u}\right)\cdot W$$

This formulation highlights the combination of individual node representations with a summation operation, followed by a multiplication with the weight matrix **W**.

Few‐shot Prompt Tuning: After conducting research, it has been observed that the success of pre‐training‐prompt tasks in the NLP field can largely be attributed to the significant similarities between pre‐training and downstream tasks. These commonalities allow the knowledge acquired during pre‐training to be effectively utilized in downstream tasks.

To better adapt the designed prompt to downstream tasks, we freeze the parameters of the pre‐trained model and fine‐tune the prompt with a small amount of data, aligning the pre‐trained model more effectively with the downstream task. In order to identify the shared information between pre‐training and downstream tasks, we have established two virtual task bridges, $B_{T}$ and $B_{F}$, representing positive and negative node instances during the few‐shot prompt tuning step:

(9) $$B_{T}=\frac{1}{n}\sum_{\begin{array}{c} v_{i}\in T \end{array}}s_{v_{i}}\;\;,\;\;B_{F}=\frac{1}{n}\sum_{\begin{array}{c} v_{i}\in F \end{array}}s_{v_{i}}$$

where T represents the training dataset of positive node instances, and F represents the training dataset of negative node instances. In the few‐shot scenarios, each of these sets contains only *k*% nodes, and each node consists of two entities with known interactions. Therefore, we design the following loss function to fine‐tune the downstream tasks:

(10) $$L_{\mathrm{prompt}}\left(p^{\left(z\right)}\right)=-\sum_{\left(x_{i},y_{i}\right)\in V^{\left(z\right)}}\ln\frac{\exp(sim\left(s_{x_{i}}^{\left(z\right)},B_{y_{i}}^{\left(z\right)}\right)/\tau )}{\sum_{C\in (T,F)}\exp\left(sim\left(s_{x_{i}}^{\left(z\right)},B_{C}^{\left(z\right)}\right)/\tau \right)}$$

For a specific task *z*, we define $V^{\left(z\right)}=\left\{\left(x_{1},y_{1}\right),\left(x_{2},y_{2}\right),\text{…}\right\}$ as the training set, where *x* represents a node, and *y* represents the label of *x*. In drug association prediction tasks, for instance, DTI, *x* represents the combination of a drug and a target, and *y* represents whether they have interactions. $B_{y_{i}}^{\left(z\right)}$ represents virtual task bridges, where *y*_*i*_ indicates the positive or negative label for a specific task *z*. $B_{C}^{\left(z\right)}$ denotes virtual task bridges of both positive and negative samples. It's important to note that, at this stage, adjustments are made only to the prompt vector **p** ^(*z*)^. We freeze the parameters from the pre‐training phase to enhance efficiency, reducing reliance on labeled data, and making it more suitable for few‐shot learning scenarios.

### Experimental Setup

To evaluate the performance of our model in handling downstream multi‐task scenarios with limited samples, we conduct the pre‐training process separately on two datasets. During the pre‐training of the model, we pair the nodes in the dataset based on the inherent interaction types, selecting 10,000 pairs for each interaction to construct the heterogeneous network. Note that during the pre‐training stage, we did not utilize any known interaction information. For specific tasks, we utilize the same pre‐trained model and specific prompt vectors. During testing, we employ a particular prompt vector to perform node classification. In our model, the GNN encoding layer is set to 3, with a weight decay coefficient of 1e‐4. The dimensionality of the prompt vector is set as the product of the GNN hidden dimension and the number of GNN layers, which ensures consistency with the hierarchical representation structure of the encoder and provides sufficient capacity for downstream adaptation. In all experiments, we applied 10‐fold cross‐validation. In terms of computational cost, the floating‐point operations (FLOPs) required for pre‐training amount to approximately 1.687 GFLOPs. For each downstream fine‐tuning task (excluding pre‐training), the model requires approximately 3840 FLOPs, indicating a relatively low computational burden and enabling efficient deployment in resource‐constrained environments. For downstream tasks, we adopt a few‐shot setting, meaning that only *k*% samples are used for fine‐tuning the model for each task. Additionally, to balance positive and negative examples in the fine‐tuning data, we randomly shuffle an equal number of negative examples each time, i.e., meaning that we use *k*/2% positive and *k*/2% negative samples. We evaluate our model using the metric of accuracy. For all mentioned baseline methods, we adhere to using the original parameters and code from the respective papers in our experiments on the same datasets.

### Dataset

Our experiments are conducted on two publicly available datasets that include a variety of drug association relationships. **Luo's data** ^[^ ^14^ ^]^ consists of four entity types: 708 drugs, 1512 proteins, 5603 diseases, and 4192 side effects. The dataset comprises six types of interactions: drug–protein, drug–drug, drug‐disease, drug‐side effect, protein‐protein, and protein‐disease interactions (**Table** **3**). **Zheng's data** ^[^ ^35^ ^]^ includes six entity types: 1,094 drugs, 1,556 target proteins, 738 drug substitutes, 881 chemical structures, 4,063 drug side effects, and 4,098 gene ontology. The dataset comprises six types of interactions: drug‐chemical structure, drug‐drug side effect, drug‐drug substitute, drug‐target protein, and target protein‐gene ontology interactions (**Table** **4**).

**Table 3:** Statistics of Luo's dataset.

Luo's data					
Node		Edges			
node	node	Drug‐	Drug‐	Drug‐	Protein‐
number	type	protein	disease	side effect	disease
12,015	4	1922	199,214	80,164	1,596,745

**Table 4:** Statistics of Zheng's dataset.

Zheng's data						
Node		Edges				
node	node	Drug‐	Drug‐	Drug‐	Drug‐	Target‐
number	type	chemical	side effect	substituent	target	go
12,430	6	133,880	122,792	20,798	10,819	35,980

### Main Comparison Methods‐GCN

GCN (Graph Convolutional Network)^[^ ^36^ ^]^ is used for extracting information from graph data. It primarily utilizes the graph's adjacency matrix and feature matrix, employing linear transformations and nonlinear activation functions to obtain low‐dimensional vector representations for each node, which are then applied in downstream tasks.

### Main Comparison Methods‐GAT

GAT (Graph Attention Network)^[^ ^37^ ^]^ is a graph neural network that incorporates attention mechanisms. It utilizes multiple graph networks to learn low‐dimensional representations of nodes and edges. The model employs self‐attention mechanisms to identify optimal mappings and predict relationships between nodes.

### Main Comparison Methods‐GraphSAGE

GraphSAGE^[^ ^34^ ^]^ is an inductive framework for extracting graph data, which effectively leverages node attribute information to learn embeddings for newly encountered nodes. Its central concept involves the acquisition of an aggregation function, such as averaging or maximum pooling, to merge neighborhood information of nodes into their own features, thereby obtaining low‐dimensional vector representations for the nodes.

### Main Comparison Methods‐GPPT

GPPT^[^ ^27^ ^]^ introduces a novel transfer learning paradigm for the generalization of Graph Neural Networks (GNN). The core concept involves pretraining a GNN by leveraging a task focused on predicting masked edges. Following this, a graph prompt function is used to reframe downstream tasks, aligning them with the pretraining task to narrow the task gap. The approach introduces methods for generating task and structure tokens, facilitating the creation of node prompts for node classification tasks.

### Main Comparison Methods‐DGI

DGI^[^ ^38^ ^]^ framework is designed for unsupervised representation learning of graph‐structured data, efficiently utilizing node attribute information to embed newly encountered nodes. At its core, it employs Graph Convolutional Networks (GCN) to generate local features for nodes and global features for the entire graph. The training process involves maximizing the mutual information between these features. This approach enables the model to learn node vectors that capture both the graph structure and node attributes, thereby enhancing the performance of downstream tasks, such as node classification.

### Main Comparison Methods‐GCC

GCC^[^ ^39^ ^]^ is a self‐supervised graph neural network pre‐training framework designed to learn transferable structural representations from heterogeneous graphs. Drawing inspiration from pre‐training paradigms in natural language processing and computer vision, GCC formulates a contrastive learning objective based on subgraph instance discrimination, both within and across different graph structures. This approach enables the model to capture universal topological patterns and produce graph representations that generalize effectively to out‐of‐distribution tasks and unseen domains.

### Main Comparison Methods‐Graphcontrol

GraphControl^[^ ^40^ ^]^ is a recently proposed method aimed at improving domain transferability in pre‐trained graph models by addressing the “transferability ‐ specificity dilemma”. While conventional self‐supervised graph pre‐training captures domain‐invariant structural knowledge, it often neglects task‐ or domain‐specific node attributes crucial for downstream adaptation. GraphControl mitigates this limitation by aligning the input spaces of source and target graphs and conditionally integrating task‐specific features during fine‐tuning or prompt tuning. This is achieved through a progressive conditioning mechanism that adaptively injects target‐specific signals, thereby enabling personalized and context‐aware knowledge transfer.

## Conflict of Interest

The authors declare no conflict of interest.

## Author Contributions

G.W. conceived the work, assisted in algorithm design and implementation. Y.L. and Y.S. contributed to algorithm design, implementation, and computational experiment analysis. X.Q. contributed to computational experiment analysis and visualization. X.G. contributed to algorithm design. Y.L. drafted the initial manuscript. G.W. and Y.L. contributed to manuscript revision. Y.S. contributed to data acquisition and processing.

## Data Availability

All relevant data and source codes including the constructed network and the pre‐trained graph model, can be downloaded from https://github.com/catly/MGPT.
